# Role of Lipoproteins in the Pathophysiology of Breast Cancer

**DOI:** 10.3390/membranes12050532

**Published:** 2022-05-19

**Authors:** Santhi Latha Pandrangi, Prasanthi Chittineedi, Rajasekhar Chikati, Juan Alejandro Neira Mosquera, Sungey Naynee Sánchez Llaguno, Gooty Jaffer Mohiddin, Suseela Lanka, Sphoorthi Shree Chalumuri, Narendra Maddu

**Affiliations:** 1 Onco-Stem Cell Research Laboratory, Department of Biochemistry and Bioinformatics, GITAM School of Science, GITAM Deemed to Be University, Visakhapatnam 530045, India; pchittin@gitam.in (P.C.); sphoorthichalumuri98@gmail.com (S.S.C.); 2 Department of Biochemistry, Yogivemana University, Kadapa 516005, India; chikati.rajasekhar@yvu.edu.in; 3 Department of Life Sciences and Agriculture, Armed Forces University-ESPE, Santo Domingo 230101, Ecuador; janeira1@espe.edu.ec (J.A.N.M.); snsanchez@espe.edu.ec (S.N.S.L.); jgooty@espe.edu.ec (G.J.M.); 4 Faculty of Industry and Production Sciences, Quevedo State Technical University, km 11/2 via Santo Domingo, Quevedo 120301, Ecuador; 5 Department of Biosciences and Biotechnology, Krishna University, Machilipatnam 521004, India; susheelalankaku@gmail.com; 6 Department of Biochemistry, Sri Krishnadevaraya University, Anantapur 515003, India; narendramaddu@gmail.com

**Keywords:** cholesterol, lipoproteins, breast cancer, metastasis

## Abstract

Breast cancer is one of the most common malignancies in women and the leading cause of cancer mortality. Hypercholesterolemia and obesity are potential risk factors for the incidence of breast cancer, and their detection can enhance cancer prevention. In this paper, we discuss the current state of investigations on the importance of lipoproteins, such as low denisity lipoproteins (LDL) and high density lipoproteins (HDL), and cholesterol transporters in the progression of breast cancer, and the therapeutic strategies to reduce breast cancer mortality. Although some research has been unsuccessful at uncovering links between the roles of lipoproteins and breast cancer risk, major scientific trials have found a straight link between LDL levels and incidence of breast cancer, and an inverse link was found between HDL and breast cancer development. Cholesterol and its transporters were shown to have significant importance in the development of breast cancer in studies on breast cancer cell lines and experimental mice models. Instead of cholesterol, 27-hydroxycholesterol, which is a cholesterol metabolite, is thought to promote propagation and metastasis of estrogen receptor-positive breast cancer cell lines. Alteration of lipoproteins via oxidation and HDL glycation are thought to activate many pathways associated with inflammation, thereby promoting cellular proliferation and migration, leading to metastasis while suppressing apoptosis. Medications that lower cholesterol levels and apolipoprotein A-I mimics have appeared to be possible therapeutic agents for preventing excessive cholesterol’s role in promoting the development of breast cancer.

## 1. Introduction 

Breast cancer is one of the most common cancers, having a high incidence among female patients [1]. Lack of physical exercise, a high-fat diet, and alcohol consumption are some of the major reasons for the development of mammary gland malignancies. Typically, breast cancers are classified into seven subtypes: luminal A, which is characterized by histologic low-grade malignancies and is ER-positive; luminal B is characterized by histologically high-grade malignancies and is ER-positive; the human epidermal growth factor receptor-2 (HER2) overexpression type of breast cancer—immunomodulatory (IM), basal-like (BL1 and BL2), mesenchymal (M), and mesenchymal stem-like (MSL) breast cancer [2], and normal breast-like tumors fall under this category [3]. The majority of triple-negative breast cancers (TNBCs) which do not express estrogen receptor (ER), progesterone receptor (PR), and HER2 are basal-like, and many basal-like breast tumors are triple-negative; nevertheless, they are not equivalent in terms of gene expression profiles and immunohistochemical analyses [4]. Basal-like breast carcinoma is a subtype of breast cancer defined by its gene expression profiles. Even though they seem to be comparable, there is up to 30% conflict between both groups [5,6,7,8]. Furthermore, basal-like breast tumors have elevated expression of CK5, CK14, caveolin-1, caix, p63, and EGFR (epidermal growth factor receptor)/HER1; and the reduced expression of ER, PR, and HER2 impacts the basal/myoepithelial cell component of the mammary gland.

Cancer cells exhibit distinct abnormalities in several areas of lipoprotein absorption, which can influence the availability of the metabolized lipids for membrane production, the lipid contribution to energy balance, and lipid signaling activities, such as the stimulation of inflammation-related cascades [9,10]. All of these alterations are associated with critical biological activities, such as cellular metabolism, differentiation, progression, and cellular motility. The interactions of lipoproteins, cholesterol, pro-inflammatory signal transduction, and cancer progression have been explored primarily in breast cancer cells and in vivo studies [11,12]. Additionally, despite the lack of epidemiological evidence, both benign and malignant tumors’ tissue growth has been related to alterations in plasma lipoprotein and lipid levels in people. The relationship between lipoproteins and risk of breast cancer incidence yielded ambiguous findings [13].

Cholesterol is an amphipathic sterol biomolecule that is essential for the maintenance of biological equilibrium. It is slightly complex molecule comprising 27 carbon moieties that begin with 2-carbon components [14]. This alone demonstrates the significance of this biomolecule. Cholesterol is a precursor of various metabolites, such as bile acids; vitamin D; and hormones such as androgens, progestogens, estrogens, and corticosteroids. Elevated cholesterol, while necessary, is linked to cardiac diseases and renal illness, and cancer onset, recurrence, and metastasis. Recent research reveals links between the risk and degree of cancer incidence and circulating cholesterol levels.

Estrogens are implicated in a lot of biological mechanisms, including energy homeostasis, stress reactions, mineral balance, and sexual development. In premenopausal women, estrogens are primarily produced by the ovary [15]. The hypothalamus stimulates follicle-stimulating hormone (FSH) and luteinizing hormone (LH) by secreting gonadotropin-releasing hormone (GnRH). FSH enhances the secretion of estrogen in developing ovarian sacs, which subsequently operates on the brain to generate LH production [16]. The ovaries generate insignificant quantities of estrogen after menopause. The fact that early menstruum and menopause at a very old age might significantly contribute to breast cancer emphasizes the relevance of steroidogenesis in the gonads to normal breast development and the emergence of breast cancer. Similarly, menarche at very young age and menopause before the age of 40 reduce the chance of getting breast cancer significantly. As a result, it is somewhat puzzling that the majority of breast malignancies arise in postmenopausal women with low levels of circulating estrogen [17].

Obesity, on the other hand, has been progressively increasing over the world, and there is a good evidence to show a causal relationship between obesity and onset of numerous malignancies, including ovarian, breast, pancreatic, and renal cancers; multiple myeloma; leukemia; and esophageal cancer [18]. Development of ER+ breast cancer in postmenopausal women showed a greater correlation, indicating that estrogens play a key role in promoting adiposity, thereby contributing to the development of breast cancer. Interestingly, in postmenopausal women, the body’s principal source of estrogen production is the adipose tissue. Surprisingly, BMI has been discovered to be associated with tissue estrogen levels in a positive manner [19]. As a result, as body mass increases, expression of aromatase results in a subsequent rise in estrogen levels, a consequence that is particularly pronounced in postmenopausal women [20]. In this article, we focus on the significance of lipoproteins in breast cancer invasion, the prime role of adiposity in breast cancer development, and the therapeutic strategies targeting lipoprotein metabolism to combat breast cancer progression.

### 1.1. Role of Obesity in Breast Cancer Development

Obesity has been associated with elevated risks and incidences of several malignancies. Chronic inflammation in adipocytes, which results in genotoxic stress, may have a role in carcinogenesis and cancer onset. The evidence that adipose tissue has a role in tumor progression is emerging. Malignant cells have a metabolically synergetic relationship with neighboring adipose tissue as the cancer cells proliferate [21]. Mature adipocytes supply lipids and adipokines to the tumor cells, whereas immune cells and stromal cells from adipose tissue infiltrate malignant cells and release paracrine signals locally in the tumor microenvironment. A plethora of strategies has been hypothesized to explain obesity’s influence on cancer incidence and its progression [22]. Chronic inflammation, hyperinsulinemia; and alterations in serum steroid hormone levels, glucose, and lipids, along with cytokines and growth factors, including IGF-1, leptin, and adiponectin, are examples of these [23]. A complete description of these circulating chemicals, and their roles in cancer, has already been published. While nutrition is crucial when examining the obesity–cancer relationship, animal studies suggest that white adipose tissue expansion enhances tumor progression directly, regardless of diet.

### 1.2. Role of Adiposity in Obese Patients and Its Negative Effect on Prognosis

Adipose tissue is an important tissue in the endocrine system that has a role in both obesity and cancer onset. It is commonly associated with excess fat in the body, and it is widely known that female breast tissue has a lot of it [24]. Adipose tissue (AT) is composed of adipocytes, immune cells, and an extracellular matrix (ECM), which play important roles in breast changes during a female breast’s life cycle, including puberty, pregnancy, breastfeeding, and involution [25]. When there is a need for energy supply, TAG stored in adipocytes is released as fatty acids to serve other tissues during fasting or periods of high energy demand [26]. As a result, AT plays a vital role in the control of systemic lipid metabolism, and dietary and hormonal signals help to balance lipid accumulation and breakdown inside the fat cell. 

Adipose tissue has three different types: white AT, brown AT, and beige AT. The largest energy storage compartment is white adipose tissue (WAT), which consists of cells with large cytoplasmic lipid drops. It provides energy in between food consumption. It is also known to create a large number of pro-inflammatory chemicals and a large number of adipokines connected with inflammatory alterations, and to have poor metabolic activity [27]. Brown adipose tissue (BAT) was thought to be found only in hibernating organisms and infants. However, it has been found in small reserves around the neck and the interscapular area. Small lipid droplets, large numbers of capillaries, and iron-rich, large, spherical, and packed mitochondria that are employed to deliver oxygen to BAT for energy production and for the dissemination of energy to the remaining body characterize BAT [28]. Beige/brite (brown-like) adipose tissue has a significant role in both storing energy and thermogenesis.

Several studies have shown that breast adipose tissue has a function in the development of mammary glands. Breast adipose tissue is an important part of the breast’s endocrine system, secreting a variety of growth factors and enzymes [25]. It has been proven in vitro to have a function in mammary epithelial cell development. Studies using breast stromal cells in a co-transplantation design revealed that mammary adipose tissue is crucial for the typical development of epithelial cells in the breast [29].

### 1.3. Importance of Cholesterol in Lipoprotein Synthesis

Adipose tissue, which is regarded as a fat storage moiety, plays a crucial role in the pathophysiology of breast carcinoma by providing all the required nutrients to the tumor microenvironment. Cholesterol, a 27-carbon amphiphilic lipid molecule, stabilizes and regulates cell membrane fluidity and permeability regardless of temperature changes. Cholesterol, together with sphingolipids, phospholipids, and glycosylphosphatidylinositol-anchored proteins, plays a crucial role in the formation and stabilization of lipid microdomains known as lipid rafts [11,14,30]. Several studies have found that numerous oncogenic signaling pathways affect cholesterol production, implying that they play a role in tumorigenesis. Cholesterol is transported via lipoproteins, which act as transporter molecules. Cholesterol is a hydrophobic, non-polar substance that could not pass through the bloodstream. Lipoproteins have an amphipathic surface layer of free cholesterol and phospholipids. Lipoproteins are made up of different combinations of hydrophobic triglyceride and cholesterol ester molecules. Lecithin cholesterol acyltransferase (LCAT), an enzyme located in the peripheral tissues, and ACAT2 in enterocytes bordering the intestinal lumen, result in the production of hydrophobic cholesterol ester from free cholesterol; this allows storage of more cholesterol molecules in individual lipoproteins [31,32].

The many distinct lipoproteins involve various soluble proteins that combine with and transport the cholesterol to their destinations; they are classed in terms of the concentrations, relative size, and associations with apolipoproteins [33]. HDL, intermediate-low density lipoproteins (IDL), LDL, very-low density lipoproteins (VLDL), and chylomicrons are the traditional categories [34]. Lipoprotein (a) is another lipoprotein that is an LDL moiety with an additional apolipoprotein (a) (apo (a) connected to the LDL particle’s apolipoprotein B-100 (ApoB-100) component through a disulfide bridge). Additionally, lipid-containing exosomes also play a crucial role in cancer metastasis [35]. Table 1 represents various types of lipoproteins and their properties.

LDL serves to lodge cholesterol into damaged cells. The liver may also endocytose LDL particles, releasing the cholesterol and fat components for their metabolism and excretion as well. The majority of lipoproteins are toxic to cells, which means they stimulate the development of atherosclerotic plaques [40]. However, HDL is not atherogenic; it may associate with both LDL and chylomicrons, and adipose tissue and muscle cells, to obtain cholesterol for transit back to the liver, where it can be expelled as bile [41].

## 2. Clinical and Epidemiologic Studies of Breast Cancer Incidence in Association with Cholesterol Levels

The investigation of the associations between circulating cholesterol levels and cancer incidence is of particular interest and has caused discussion, particularly with the introduction of lipid-modulating medications and antagonistic cholesterol targets to minimize the risks for various heath disorders. However, several studies have yielded contradictory findings. Indeed, one study discovered that total circulating cholesterol levels are connected with the risk of breast carcinoma development, whereas other studies were unsuccessful in uncovering such a relationship, or discovered that total cholesterol was negatively associated with higher severity of breast cancer [31]. As LDL and HDL are the primary carriers of cholesterol, multiple clinical investigations have linked them to malignancy in the breast. A clinical investigation that examined the lipid profiles in females with carcinoma in the breast discovered that circulating LDL cholesterol (LDL-C) levels could be a predictive factor for breast tumor growth [11]. 

A LDL-C level of more than 117 mg/dL was thought to be a predictor of cancer stage, and it is associated with the worst prognosis, due to its association with cancer grade, proliferative rate, and a more progressive clinical stage. Furthermore, those with circulating LDL-C levels exceeding 144 mg/dL are more likely to have lymph node metastases [40]. More crucially, studies conducted using Mendelian randomization discovered that elevated circulating LDL-C due to hereditary factors was linked to a high incidence of breast carcinoma. Disagreements in HDL-C levels were also discovered. One prospective study with an 11.5-year follow-up period discovered a contrary relationship between circulating HDL-C and risk of acquiring breast cancer, and retrospectively acquired data from clinical samples revealed that lower circulating HDL-C levels were associated with poor overall survival in women with breast carcinoma [42]. Mendelian randomization research, on the other hand, found that high HDL-C levels amplified the risk of estrogen receptor (ER)-positive breast carcinoma [38]. Other studies, it should be mentioned, have shown no link between circulating HDL-C and breast cancer incidence or survival. Furthermore, there is disagreement when it comes to patients’ menopausal state.

### 2.1. LDL and Breast Cancer

Tumor cells that are proliferating would have an elevated requirement for cholesterol. LDL-receptor overexpression in histopathology was found to promote LDL-C absorption from circulation. In contrast with ER-positive MCF-7 cell lines, gene and protein expression of LDLR was upregulated in ER-negative MDA-MB-231 cell lines in vitro [30,43]. As a result, LDL-C mostly boosted proliferation and invasion in ER-ve cell lines, but not in ER+ve cell lines. Due to the enhanced enzyme activity of acyl-CoA—cholesterol acyltransferase 1 (ACAT1)—ER-negative cells have a better capacity to take in, store, and use exogenous cholesterol. The Women’s Intervention Nutrition Study (WINS) discovered that a less fatty diet significantly increased survival in ER-negative breast cancer patients, and associated with a decreased rate of cancer relapse in the breast [44]. The fact that ER-ve breast cancer cell lines ingest and preserve cholesterol differently might explain, at least in part, why a less-fatty diet has a distinct effect on human breast tumor recurrence. In another investigation, LDL-C was observed to stimulate the ER+ve breast cancer cell line BT-474’s proliferation. This disparity might be explained by the fact that BT-474 cell lines frequently express the Her2 (ErbB2) receptor, and high circulating plasma LDL-C levels have been linked to Her2+ve breast cell lines. It is worth noting that the Her2+ve and TNBC subtypes of breast carcinoma show high proliferation rates [45].

LDL-C elevation has been linked to a tumor size of about 20 mm and lymph node metastases. Interestingly patients with >144 mg/dL circulating cholesterol levels showed HER2 positivity. LDL-C also enhances breast cancer cell motility by reducing claudin and occludin (adhesion proteins) expression [46]. Cellular inflammation and damage in association with elevated intracellular ROS levels might be a possible mechanism for LDL-promoted carcinogenesis. LDL-C can activate HER2, through activation of ERK and Akt pathways, which results in aggressive cellular proliferation in tumor cells [47]. Phosphorylation of the FOXO3a by ERK triggers its degradation and thereby prevents cell cycle arrest by inducing apoptosis by triggering Bim and FasL activation. It is required for proper regulation of the cell cycle via inducing p27kip1 and cyclin D [48]. As a result, ERK-mediated FOXO3a degradation could enhance cellular survival and proliferation via cell cycle dysregulation. Akt activation, on the other hand, affects cell survival in a variety of ways. Together with p53 gene knockout, enhanced Bcl-xL expression, and activated mTOR signaling, it could also drive cell proliferation by suppressing p21 and p27 [49].

### 2.2. HDL and Breast Cancer

HDL is the primary lipoprotein accountable for carrying cholesterol to the liver from the peripheral for elimination. Apolipoproteins A1, A2, C-1, and E are apolipoproteins related to HDL. Apo-A1 and Apo-A2 are the most common apolipoproteins that trigger the biosynthesis of HDL [40]. ApoA1, which is largely generated in the small intestine and in the liver, is expressed in the majority of HDL molecules, and accounts for approximately 70% of their lipoprotein composition. Apo-A2 is generated only in the liver and accounts for about 20% of their lipoprotein composition; it is expressed on around 67 percent of HDL molecules [34]. When apo-A1 is released, it is lipidated by triglycerides and free cholesterol, resulting in the formation of precursor HDL molecules. The liver and intestine are principally responsible for early lipidation, as they produce these essential lipids via the ABCA1 transporter. During this phase, the ABCA1 transporter may also extract cholesterol and phospholipids from macrophages [49]. HDL also obtains a large quantity of cholesterol from extrahepatic tissues that are unable to process cholesterol on their own [8,31,40]. 

This cholesterol transport is mediated by several pathways. Significant cholesterol transfer is mediated by aqueous, passive diffusion across cellular membranes and HDL molecules, which is aided by the activation of the ABCG1 transporter, which effluxes cholesterol from organelles to boost passive diffusion [49]. Passive, non-aqueous cholesterol transport can also be mediated by scavenger receptor BI proteins (SR-BI). The development of cholesterol esters, which could increase the HDL core, marks the maturity of HDL molecules from their primordial forms. Apo-A1 triggered LCAT activation is used in this esterification. LCAT’s capacity to synthesize cholesterol esters from free cholesterol improves HDL’s ability to transport cholesterol.

In breast cancer cells, SR-BI functions as a receptor for HDL and facilitates absorption of free cholesterol. When compared to nearby normal tissue, the SR-BI receptor is abundantly expressed in human breast tumor tissue [50]. Furthermore, upregulation of SR-BI was found to be associated with greater tumor progression and a poor prognosis in breast carcinoma, whereas in vitro SR-BI knockdown studies showed a reduction in Akt activation, thereby hindering breast cancer aggression and metastasis. Furthermore, HDL-induced proliferation was inhibited in MCF-7 cells transfected with a mutant, non-functional SR-BI [51]. In addition to in vitro investigations, in vivo studies conducted in mice suggested a good survival rate with a low tumor burden, which was associated with decreased Akt and ERK1/2 activation, decreased angiogenesis, and enhanced cell death [50].

## 3. Clinical and Epidemiological Studies of the Roles of Lipoproteins in Various Malignancies

Studies done by Jamnagerwalla et al. suggest an association between total circulating cholesterol, HDL-C, and LDL-C; and observed that high circulating cholesterol along with increased HDL-C were positively correlated with an elevated risk of high-grade prostate cancer. 

In 2015, Yang et al. conducted a study among 39 patients with hematological malignancy and 19 healthy volunteers to find the association between the incidence of hematological malignancy with respect to the levels of oxidized LDL. The results suggest that patients diagnosed with hematological cancer had elevated levels of oxidized LDL.

Similarly to Yang et al., Diakoswaka et al. have also evaluated the levels of oxidized LDL in 73 patients diagnosed with colorectal cancer and 30 healthy volunteers. Although the sample sizes of both studies were small, they suggest that elevated oxidized LDL is a risk factor for leukemia and colorectal cancer. Interestingly Diakoswaka et al. observed that levels of oxidized LDL were significantly higher in the early stage of colorectal cancer compared with advanced stages, suggesting that oxidized LDL could be used to predict the risk of colorectal cancer at an early stage. 

## 4. Cholesterol-Lowering Therapies for Breast Cancer

**Figure 1:**
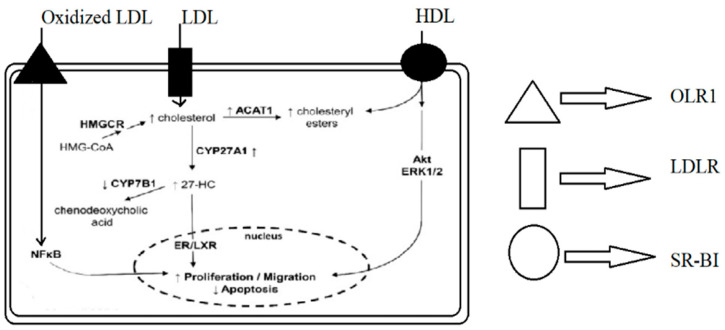
**Mechanisms by which lipoproteins and their modified forms induce proliferation and migration and reduce apoptosis in breast cancer cells**. OLR1—OxLDL lecithin-like receptor 1, LDLR—LDL receptor, SR-BI—scavenger receptor class B type I, HMGCR—hydroxy-methyl-glutaryl-coenzyme A reductase, ACAT1—acetyl-CoA cholesterol acyltransferase 1, 27-HC—27-hydroxycholesterol, ERK1/2—extracellular signal-regulated kinases ½, NFκB—nuclear factor kappa-B, and ER/LXR—estrogen receptor/liver X receptor.

According to the research examined, circulating cholesterol and its primary metabolite, 27-HC, might facilitate the growth and progression of breast cancer. To address this, medications that lower circulating cholesterol have been developed as promising therapies for reversing the negative consequences of poor lipoprotein metabolism in malignancy. Figure 1 demonstrates the mechanism of lipid metabolism and its impact on apoptosis.

**Figure 2:**
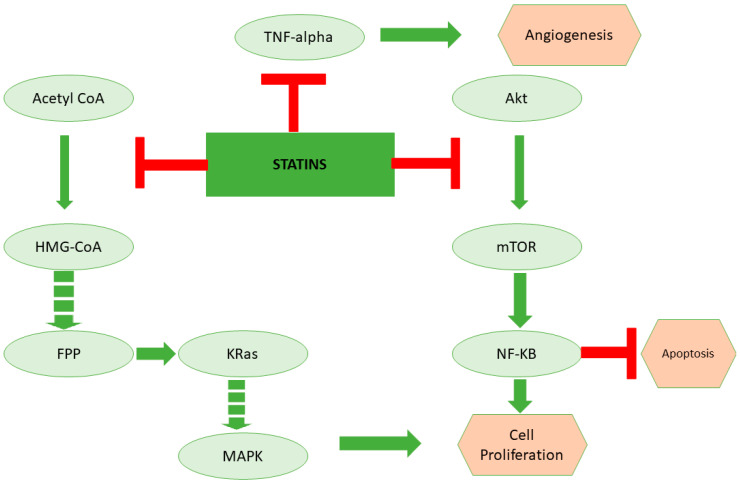
**The role of statins in inducing cancer cell death through inhibition of lipoprotein metabolism.** Statins are the cholesterol-lowering drugs that inhibit the mevalonate pathway. Apart from cholesterol biosynthesis, the mevalonate pathway is the key regulator for the synthesis of kRas, which is a critical regulator of the cell cycle. On the other hand, studies suggest that statins could also potentially inhibit TNF-alpha and Akt, which are important for angiogenesis and inhibition of apoptosis, respectively.

The use of lipid-lowering medications, specifically statins, has been linked to a decreased incidence of breast cancer in elder women by halting the synthesis of mevalonate, thereby decreasing serum LDL-C, triglycerides, and cholesterol levels [52]. On the other hand, lipophilic statins were found to dramatically lower the incidence of breast malignancy in Thai people [35,53,54]. Other investigations, including major Mendelian randomization research, revealed no ability of statins to reduce breast cancer risk, nor did they even identify a positive connection between long-term statin usage and an elevated risk of breast cancer. However, these studies had short follow-up times, and further investigation is needed to find the correlation of reduced breast-cancer risk with long-term usage of statins [55]. Statin therapy, on the other hand, appears to be more legitimate in halting breast cancer recurrence and mortality. In terms of statin type, lipophilic statins were shown to be related to effectively reducing the risk of breast cancer recurrence or death, whereas hydrophilic statin usage was also correlated with enhanced progression-free survival in inflammatory breast cancer patients when compared to no statin use [56]. Hydroxy-methyl-glutaryl-coenzyme A reductase (HMGCR) inhibitors does not appear to prevent the incidence of breast cancer when taken jointly; however, statins, and more specifically, lipophilic statins, might be a viable approach to protect breast cancer diagnosed patients against breast cancer relapse and mortality [53]. Statins are also cytotoxic and have antiproliferative properties against breast cancer cell lines in vitro, promoting autophagy, apoptosis, and cell cycle arrest [57]. Only lipophilic statins, however, have an anticancer effect, and the ER-ve phenotype appears to be more susceptible than those that overexpress ER. High expression of cholesterol biosynthesis genes is related to ER+ve cell resistance to statin therapy [53,58]. Figure 2 demonstrates the role of statins in inducing cancer cell death through inhibition of lipoprotein metabolism.

Ezetimibe, on the other hand, is a medication that inhibits intestinal sterol absorption by directly targeting Niemann-Pick C1-like 1 (NPC1L1). Few studies have been conducted to examine the impact of ezetimibe on breast carcinoma. Given that statins might not affect circulating plasma cholesterol in mice, ezetimibe’s effect on tumor growth is fascinating [59]. Pelton et al. studied the effects of ezetimibe in an HFHC regime on the development of breast carcinoma in an orthotopic malignant lesions model in which mice were implanted with the MDA-MB-231 cell line [45,60]. When compared to HFHC-fed animals, ezetimibe reduced tumor size and proliferation; inhibited angiogenesis; and increased caspase activity, yielding effects comparable to those seen in mice given a less-fatty/low-cholesterol (LFLC) diet. These findings were followed by a decrease in blood cholesterol levels, but not in intratumoral cholesterol levels [61].

## 5. Future Possibilities of Targeting Lipid Metabolism to Reduce Breast Cancer Risk

There are several novel drugs, such as PCSK9 inhibitors, ANGPTL3 inhibitors, endothelial lipase inhibitors, and HDL mimetics, that have potential roles in regulating lipoprotein levels. PCSK9 attaches to LDL receptor and modulates cholesterol metabolism. Previous literature supports the importance of PCSK9 inhibition in inducing cell death in various malignancies. Khaldoun et al. were the first to report the attenuation of cancer cell progression and tumor recurrence in breast cancer via targeting the PCSK9–LDL receptor axis [62]. In vitro studies demonstrated that pseurotin A (PS) downregulated PCSK9 expression in a dosage-dependent manner and caused a concomitant elevation in LDLR in breast cancer malignancy [63]. Secondly, endothelial lipase, which is a crucial enzyme for regulating lipoprotein metabolism, intracellular lipid composition, and cytokine expression, is thought to play an important role in cancer cell metabolism via supplying fatty acids to the tumor cells that are required for the tumor cell progression [64]. Hence, inactivating endothelial lipase might serve as potential target, to induce lipid starvation in tumor cells, thereby sensitizing them to cell death mechanisms. On the other hand, ANGPTL3, the lipoprotein lipase inhibitor, serves as one of the major risk factor contributing to increased HDL-C and triglycerides and is overexpressed in various malignancies [65]. However, little is known about the significance of lipoprotein inhibitors to reducing the risk of breast cancer. To the best of our knowledge, we believe that inhibiting lipoprotein synthesis would yield good prognosis in patients diagnosed with breast cancer.

## 6. Conclusions

The findings of certain major clinical studies imply a direct link between LDL-C and breast cancer risk, and an inverse association between circulating HDL-C and risk of developing breast cancer; however, these conclusions have not been replicated in other epidemiologic investigations and are currently being contested. Basic research investigations have established the importance of cholesterol, particularly the 27-HC metabolite, and its transporters in the development of malignancy in breast [38]. Both circulating LDL and HDL may induce breast cancer through a variety of methods. Both in vitro and in vivo experimental models of breast carcinoma have revealed a link between changed proinflammatory signaling pathways, lipoproteins, and tumorigenic processes in breast carcinoma. Cholesterol can be esterified or converted to 27-HC, which is thought to stimulate the growth of ER+ve breast cancer cells, rather than cholesterol. Considering the significance of cholesterol in the progression of breast cancer, cholesterol-dropping medicines and apoA-I mimetics with anti-inflammatory and antioxidant properties might emerge as promising therapeutics for reducing the harmful effects of excessive cholesterol in breast cancer [34]. Lipophilic statins appear to be an effective method to abscond breast cancer recurrence and mortality [56]. More human studies are needed, however, to assess the effectiveness of alternative medicines, such as ezetimibe, phytosterols, or fibrates, in reducing breast cancer incidence and improving its prognosis.

## Figures and Tables

**Table 1:** Various types of lipoproteins and their properties.

Lipoprotein Type	Density (g/mL)	Major Lipids	Major Apoproteins	Properties
Chylomicrons	<0.930	Triglycerides	Apo B-48, Apo C, Apo E, Apo A-I, A-II, A-IV	Lowest protein-to lipid ratio; comprising about 90% of the lipid content [14]
Very low-density Lipoprotein	0.930–1.006	Triglycerides	Apo B-100, Apo C, Apo-E	Has high cholesterol content when compared with chylomicrons. Major triglyceride carrier [36].
Intermediate low-density Lipoprotein	1.006–1.019	Triglycerides Cholesterol	Apo B-100, Apo C, Apo-E	Triglyceride scavenger [37].
Low-density Lipoprotein	1.019–1.063	Cholesterol	Apo B-100	Smaller, denser, readily oxidized molecules and are associated with great atherogenicity [38].
High-density Lipoprotein	1.063–1.210	Cholesterol Phospholipids	Apo A-I, Apo A-II, Apo C, Apo-E	Inhibits oxidation, coagulation, activation of endothelium, platelet aggregation, and inflammation [31].
Lipoprotein (a)	1.055–1.085	Cholesterol	Apo B-100, Apo (a)	High affitinity towards arterial wall and exihibits thrombogenic properties [39].

## Data Availability

Not applicable.

## References

[1] Nelson E.R., Chang C.Y., McDonnell D.P. (2014). Cholesterol and Breast Cancer Pathophysiology. Trends Endocrinol. Metab..

[2] Lehmann B.D., Bauer J.A., Chen X., Sanders M.E., Chakravarthy A.B., Shyr Y., Pietenpol J.A. (2011). Identification of human triple-negative breast cancer subtypes and preclinical models for selection of targeted therapies. J. Clin. Investig..

[3] Elmore S. (2007). Apoptosis: A Review of Programmed Cell Death. Toxicol. Pathol..

[4] Freres P., Collignon J., Gennigens C., Scagnol I., Rorive A., Barbeaux A., Coucke P., Jerusalem G. (2010). Le cancer du sein “triple négatif”. Rev. Med. Liege.

[5] Bertucci F., Finetti P., Cervera N., Esterni B., Hermitte F., Viens P., Birnbaum D. (2008). How Basal Are Triple-Negative Breast Cancers?. Int. J. Cancer.

[6] Chikati R., Pandrangi L.S., Gundampati R., Vemuri S.H., Lakhanpal M., Singh S.S., Saxena S., Kuma C.S. (2018). Molecular Studies on Evaluation of Phytol as Cytoskeleton Targeting Element in Cancer. Int. J. Sci. Eng. Res..

[7] Nielsen T.O., Hsu F.D., Jensen K., Cheang M., Karaca G., Hu Z., Hernandez-Boussard T., Livasy C., Cowan D., Dressler L. (2004). Immunohistochemical and Clinical Characterization of the Basal-Like Subtype of Invasive Breast Carcinoma. Clin. Cancer Res..

[8] Lakhanpal M., Singh L.C., Rahman T., Sharma J., Singh M.M., Kataki A.C., Verma S., Pandrangi S.L., Singh Y.M., Wajid S. (2016). Study of single nucleotide polymorphisms of tumour necrosis factors and HSP genes in nasopharyngeal carcinoma in North East India. Tumor Biol..

[9] Santos C.R., Schulze A. (2012). Lipid metabolism in cancer. FEBS J..

[10] Pandrangi S.L., Chalumuri S.S., Garimella S. (2022). Emerging Therapeutic Efficacy of Alkaloids as Anticancer Agents. Ann. Rom. Soc. Cell Biol..

[11] Lane D.M., Boatman K.K., McConathy W.J. (1995). Serum lipids and apolipoproteins in women with breast masses. Breast Cancer Res. Treat..

[12] Rambatla P.K., Pandrangi S.L., Rentala S., Sireesha V. (2021). A Study on the Expression of CCL5, CXCR4 and Angiogenic Factors by Prostate Cancer Stem Cells. Ann. Rom. Soc. Cell Biol..

[13] Park J., Morley T.S., Kim M., Clegg D.J., Scherer P.E. (2014). Tumour Progression and Recurrence. Nat. Rev. Endocrinol..

[14] Luo J., Yang H., Song B.L. (2020). Mechanisms and regulation of cholesterol homeostasis. Nat. Rev. Mol. Cell Biol..

[15] Schüler-Toprak S., Weber F., Skrzypczak M., Ortmann O., Treeck O. (2021). Expression of estrogen-related receptors in ovarian cancer and impact on survival. J. Cancer Res. Clin. Oncol..

[16] Pike M.C., Spicer D.V., Dahmoush L., Press M.F. (1993). Estrogens, progestogens, normal breast cell proliferation, and breast cancer risk. Epidemiol. Rev..

[17] Lidegaard O. (2012). 28 Reproductive Factors and Breast Cancer. Maturitas.

[18] Vucenik I., Stains J.P. (2012). Obesity and cancer risk: Evidence, mechanisms, and recommendations. Ann. N. Y. Acad. Sci..

[19] Myers J. (2018). The Ecology of Modernism: American Environments and Avant-Garde Poetics. J. Mod. Lit..

[20] Zhao H., Zhou L., Shangguan A.J., Bulun S.E. (2016). Aromatase Expression and Regulation in Breast and Endometrial Cancer. J. Mol. Endocrinol..

[21] Parekh N., Chandran U., Bandera E.V. (2012). Obesity in cancer survival. Annu. Rev. Nutr..

[22] Ouchi N., Parker J.L., Lugus J.J., Walsh K. (2011). Adipokines in inflammation and metabolic disease. Nat. Rev. Immunol..

[23] Paz-Filho G., Lim E.L., Wong M.L., Licinio J. (2011). Associations between adipokines and obesity-related cancer. Front. Biosci..

[24] Miana V.V., González E.A.P. (2018). Adipose tissue stem cells in regenerative medicine. Ecancermedicalscience.

[25] Kothari C., Diorio C., Durocher F. (2020). The importance of breast adipose tissue in breast cancer. Int. J. Mol. Sci..

[26] Shi L., Tu B.P. (2015). Acetyl-CoA and the regulation of metabolism: Mechanisms and consequences. Curr. Opin. Cell Biol..

[27] Gao Z., Daquinag A.C., Su F., Snyder B., Kolonin M.G. (2018). PDGFRA/PDGFRβ signaling balance modulates progenitor cell differentiation into white and beige adipocytes. Development.

[28] Wankhade U.D., Shen M., Yadav H., Thakali K.M. (2016). Novel Browning Agents, Mechanisms, and Therapeutic Potentials of Brown Adipose Tissue. BioMed Res. Int..

[29] Sakakura T., Sakagami Y., Nishizuka Y. (1982). Dual origin of mesenchymal tissues participating in mouse mammary gland embryogenesis. Dev. Biol..

[30] Kumar G.R., Chikati R., Pandrangi S.L., Kandapal M., Sonkar K., Gupta N., Mulakayala C., Jagannadham M.V., Kumar C.S., Saxena S. (2013). Molecular docking and dynamics simulations of *A.niger* RNase from Aspergillus niger ATCC26550: For potential prevention of human cancer. J. Mol. Model..

[31] Patel K.K., Kashfi K. (2022). Lipoproteins and cancer: The role of HDL-C, LDL-C, and cholesterol-lowering drugs. Biochem. Pharmacol..

[32] Pandrangi S.L., Chittineedi P., Chikati R., Lingareddy J.R. (2022). Role of dietary iron revisited: In metabolism, ferroptosis and pathophysiology of cancer. Am. J. Cancer Res..

[33] Nakano T., Inoue I., Murakoshi T. (2019). A newly integrated model for intestinal cholesterol absorption and efflux reappraises how plant sterol intake reduces circulating cholesterol levels. Nutrients.

[34] Torres-Romero J.C., Lara-Riegos J.C., Parra E.A.E., Sánchez V.F., Arana-Argáez V.E., Uc-Colli S., Peña-Rico M.Á., Ramírez-Camacho M.A., Regalado M.D.P., Alvarez-Sánchez M.E. (2020). Lipoproteomics: Methodologies and Analysis of Lipoprotein-Associated Proteins along with the Drug Intervention.

[35] Malla R.R., Pandrangi S., Kumari S., Gavara M.M., Badana A.K. (2018). Exosomal tetraspanins as regulators of cancer progression and metastasis and novel diagnostic markers. Asia Pac. J. Clin. Oncol..

[36] Freeman M.W., Walford G.A. (2016). Lipoprotein Metabolism and the Treatment of Lipid Disorders. Endocrinol. Adult Pediatr..

[37] Dallinga-Thie G.M., Franssen R., Mooij H.L., Visser M.E., Hassing H.C., Peelman F., Kastelein J.J.P., Péterfy M., Nieuwdorp M. (2010). The metabolism of triglyceride-rich lipoproteins revisited: New players, new insight. Atherosclerosis.

[38] Cedó L., Reddy S.T., Mato E., Blanco-Vaca F., Escolà-Gil J.C. (2019). HDL and LDL: Potential new players in breast cancer development. J. Clin. Med..

[39] McCormick S.P.A. (2004). Lipoprotein(a): Biology and Clinical Importance. Clin. Biochem. Rev..

[40] dos Santos C.R., Fonseca I., Dias S., de Almeida J.C.M. (2014). Plasma level of LDL-cholesterol at diagnosis is a predictor factor of, breast tumor progression. BMC Cancer.

[41] Rader D.J. (2006). Molecular regulation of HDL metabolism and function: Implications for novel therapies. J. Clin. Investig..

[42] His M., Zelek L., Deschasaux M., Pouchieu C., Kesse-Guyot E., Hercberg S., Galan P., Latino-Martel P., Blacher J., Touvier M. (2014). Prospective associations between serum biomarkers of lipid metabolism and overall, breast and prostate cancer risk. Eur. J. Epidemiol..

[43] Lakhanpal M., Yadav D.S., Devi T.R., Singh L.C., Singh K.J., Latha S.P., Chauhan P.S., Verma Y., Zomavia E., Sharma J. (2014). Association of interleukin-1β-511 C/T polymorphism with tobacco-associated cancer in northeast India: A study on oral and gastric cancer. Cancer Genet..

[44] Iyengar N.M., Brown K.A., Zhou X.K., Gucalp A., Subbaramaiah K., Giri D.D., Zahid H., Bhardwaj P., Wendel N.K., Falcone D.J. (2017). Metabolic Obesity, Adipose Inflammation and Elevated Breast Aromatase in Women with Normal Body Mass Index. Cancer Prev. Res..

[45] Pandrangi S.L., Bagadi S.A.R., Sinha N.K., Kumar M., Dada R., Lakhanpal M., Soni A., Malvia S., Simon S., Chintamani C. (2014). Establishment and characterization of two primary breast cancer cell lines from young Indian breast cancer patients: Mutation analysis. Cancer Cell Int..

[46] Blackburn G.L., Wang K.A. (2007). Dietary fat reduction and breast cancer outcome: Results from the Women’s Intervention Nutrition Study (WINS). Am. J. Clin. Nutr..

[47] Wakeling A.E., Newboult E., Peters S.W. (1989). Effects of antioestrogens on the proliferation of MCF-7 human breast cancer cells. J. Mol. Endocrinol..

[48] Pandrangi S.L., Chittineedi P., Chalumuri S.S., Meena A.S., Mosquera J.A.N., Llaguno S.N.S., Pamuru R.R., Mohiddin G.J., Mohammad A. (2022). Role of Intracellular Iron in Switching Apoptosis to Ferroptosis to Target Therapy-Resistant Cancer Stem Cells. Molecules.

[49] Mebratu Y., Tesfaigzi Y. (2009). How ERK1/2 activation controls cell proliferation and cell death is subcellular localization the answer?. Cell Cycle.

[50] Aslam N., Nadeem K., Noreen R.J.A.C. (2015). Upregulation of scavenger receptor B1 is required for steroidogenic and non-steroidogenic cholesterol metabolism in prostate cancer. Abeloff’s Clin. Oncol. 5/E.

[51] Hoekstra M., Sorci-Thomas M. (2017). Rediscovering scavenger receptor type BI: Surprising new roles for the HDL receptor. Curr. Opin. Lipidol..

[52] Goldstein J.L., Brown M.S. (1990). Regulation of the mevalonate pathway. Nature.

[53] Sakellakis M., Akinosoglou K., Kostaki A., Spyropoulou D., Koutras A. (2016). Statins and risk of breast cancer recurrence. Breast Cancer Targets Ther..

[54] Pandrangi S.L., Chikati R., Chauhan P.S., Kumar C.S., Banarji A., Saxena S. (2014). Effects of ellipticine on ALDH1A1-expressing breast cancer stem cells-An in vitro and in silico study. Tumor Biol..

[55] Bonovas S., Filioussi K., Tsavaris N., Sitaras N.M. (2005). Use of statins and breast cancer: A meta-analysis of seven randomized clinical trials and nine observational studies. J. Clin. Oncol..

[56] Anothaisintawee T., Udomsubpayakul U., McEvoy M., Lerdsitthichai P., Attia J., Thakkinstian A. (2016). Effect of lipophilic and hydrophilic statins on breast cancer risk in thai women: A cross-sectional study. J. Cancer.

[57] Karp I., Behlouli H., LeLorier J., Pilote L. (2008). Statins and Cancer Risk. Am. J. Med..

[58] Gulati R., Naik Ramavath M., Metta V.S.M.K., Latha Pandrangi S. (2021). Exploring the CRISPR/Cas9 System in Targeting Drug Resistant Cancer Stem Cells. Ann. Rom. Soc. Cell Biol..

[59] Cedó L., Blanco-Vaca F., Escolà-Gil J.C. (2017). Antiatherogenic potential of ezetimibe in sitosterolemia: Beyond plant sterols lowering. Atherosclerosis.

[60] Pelton K., Coticchia C.M., Curatolo A.S., Schaffner C.P., Zurakowski D., Solomon K.R., Moses M.A. (2014). Hypercholesterolemia induces angiogenesis and accelerates growth of breast tumors in vivo. Am. J. Pathol..

[61] Lauridsen B.K., Stender S., Frikke-Schmidt R., Nordestgaard B.G., Tybjærg-Hansen A. (2017). Using genetics to explore whether the cholesterol-lowering drug ezetimibe may cause an increased risk of cancer. Int. J. Epidemiol..

[62] Abdelwahed K.S., Siddique A.B., Mohyeldin M.M., Qusa M.H., Goda A.A., Singh S.S., Ayoub N.M., King J.A., Jois S.D., El Sayed K.A. (2020). Pseurotin A as a novel suppressor of hormone dependent breast cancer progression and recurrence by inhibiting PCSK9 secretion and interaction with LDL receptor. Pharmacol. Res..

[63] Mahboobnia K., Pirro M., Marini E., Grignani F., Bezsonov E.E., Jamialahmadi T., Sahebkar A. (2021). PCSK9 and cancer: Rethinking the link. Biomed. Pharmacother..

[64] Yu J.E., Han S.Y., Wolfson B., Zhou Q. (2018). The Role of Endothelial Lipase in Lipid Metabolism, Inflammation, and Cancer. Histol. Histopathol..

[65] Kersten S. (2021). ANGPTL3 as therapeutic target. Curr. Opin. Lipidol..

